# Contrasting Modes of Mitochondrial Genome Evolution in Sister Taxa of Wood-Eating Marine Bivalves (Teredinidae and Xylophagaidae)

**DOI:** 10.1093/gbe/evac089

**Published:** 2022-06-17

**Authors:** Yuanning Li, Marvin A Altamia, J Reuben Shipway, Mercer R Brugler, Angelo Fraga Bernardino, Thaís Lima de Brito, Zhenjian Lin, Francisca Andréa da Silva Oliveira, Paulo Sumida, Craig R Smith, Amaro Trindade-Silva, Kenneth M Halanych, Daniel L Distel

**Affiliations:** Institute of Marine Science and Technology, Shandong University, Qingdao 266237, China; Ocean Genome Legacy Center, Department of Marine and Environmental Science, Northeastern University, Nahant, Massachusetts 01908, USA; Marine Biology and Ecology Research Centre, School of Biological and Marine Sciences, University of Plymouth, Plymouth PL4 8AA, United Kingdom; Department of Natural Sciences, University of South Carolina Beaufort, 801 Carteret Street, Beaufort, South Carolina 29902, USA; Division of Invertebrate Zoology, American Museum of Natural History, Central Park West at 79th Street, New York, New York 10024, USA; Departamento de Oceanografia, Universidade Federal do Espirito Santo, Vitória-ES, Brazil; Drug Research and Development Center, Department of Physiology and Pharmacology, Federal University of Ceará, Ceará, Brazil; Department of Medicinal Chemistry, University of Utah, Salt Lake City, Utah, USA; Drug Research and Development Center, Department of Physiology and Pharmacology, Federal University of Ceará, Ceará, Brazil; Departamento de Oceanografia Biológica, Instituto Oceanográfico da Universidade de São Paulo, São Paulo, SP, Brazil; Department of Oceanography, University of Hawai’i at Mãnoa, Hawaii, USA; Drug Research and Development Center, Department of Physiology and Pharmacology, Federal University of Ceará, Ceará, Brazil; Center for Marine Science, University of North Carolina Wilmington, North Carolina, USA; Ocean Genome Legacy Center, Department of Marine and Environmental Science, Northeastern University, Nahant, Massachusetts 01908, USA

**Keywords:** marine woodborers, shipworm, xylotrophy, xylophagy, mitochondrial gene order, deep-sea

## Abstract

The bivalve families Teredinidae and Xylophagaidae include voracious consumers of wood in shallow-water and deep-water marine environments, respectively. The taxa are sister clades whose members consume wood as food with the aid of intracellular cellulolytic endosymbionts housed in their gills. This combination of adaptations is found in no other group of animals and was likely present in the common ancestor of both families. Despite these commonalities, the two families have followed dramatically different evolutionary paths with respect to anatomy, life history, and distribution. Here, we present 42 new mitochondrial genome sequences from Teredinidae and Xylophagaidae and show that distinct trajectories have also occurred in the evolution and organization of their mitochondrial genomes. Teredinidae display significantly greater rates of amino acid substitution but absolute conservation of protein-coding gene order, whereas Xylophagaidae display significantly less amino acid change but have undergone numerous and diverse changes in genome organization since their divergence from a common ancestor. As with many bivalves, these mitochondrial genomes encode 2 ribosomal RNAs, 12 protein-coding genes, and 22 tRNAs; *atp8* was not detected. We further show that their phylogeny, as inferred from amino acid sequences of 12 concatenated mitochondrial protein-coding genes, is largely congruent with those inferred from their nuclear genomes based on 18S and 28S ribosomal RNA sequences. Our results provide a robust phylogenetic framework to explore the tempo and mode of mitochondrial genome evolution and offer directions for future phylogenetic and taxonomic studies of wood-boring bivalves.

**Significance**

Among metazoans, bivalves display unusual variation in mitochondrial genome evolution, organization, and inheritance, but the factors that influence this evolutionary lability are poorly understood. Here, we present 42 new mitochondrial genome sequences from bivalve sister clades that despite their close phylogenetic relationship and uniquely shared wood-feeding habits, differ dramatically in morphology, life history, and distribution. We show that these differences are correlated with equally dramatic differences in tempo and mode of mitochondrial genome evolution, laying the groundwork for improved understanding of the complex evolutionary interactions between marine organisms, environments, and mitochondrial genomes.

## Introduction

**Fig. 1.**
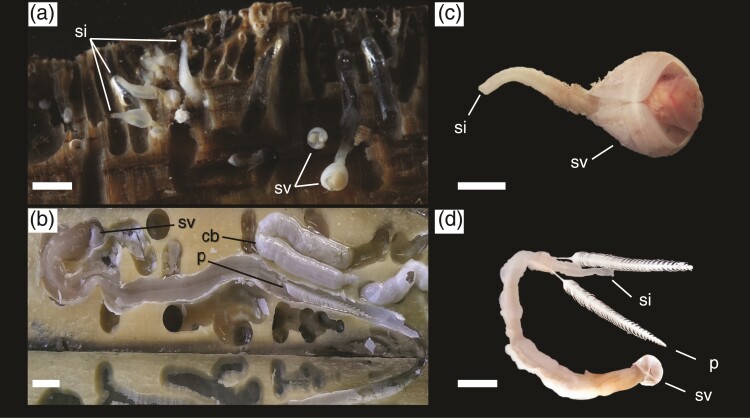
Wood-boring bivalves of the families Teredinidae and Xylophagaidae. (*a*) Xylophagaids and (*b*) Teredinids within their burrows in wood; (*c*) xylophagaid (*Xylophaga dorsalis*) and (*d*) teredinid (*Bankia setacea*) removed from their wooden burrows. Note the dramatic differences in morphology. cb, calcareous burrow lining; p, pallet; si, siphon; sv, shell valve. Scale bars for (A)–(D) = 1 cm.

Teredinidae and Xylophagaidae (fig. 1) are the sole members of the molluscan class Bivalvia capable of consuming wood as food, or xylotrophy. Indeed, all but two species of Teredinidae and all species of Xylophagaidae burrow nearly exclusively in wood or woody plant materials and are thought to utilize vascular plant cell wall material (lignocellulose) as a substantial source of dietary carbon (Distel 2003; Turner 1967; Voight 2015; Nishimoto et al. 2021). Moreover, all members of both families are thought to harbor intracellular cellulolytic gammaproteobacteria in their gill tissues, a feature that is unique among all animals. Teredinidae are commonly called “shipworms” because of their worm-like morphology and historical legacy of destroying unprotected wooden ships. Even today, shipworms account for billions of US dollars in damages to man-made wooden structures in marine and brackish environments (Distel 2003). Shipworms were well-known to the earliest seafarers and have altered the course of human civilizations (Stearns 1886; Distel 2003), having been implicated in the defeat of the Spanish Armada and in the disastrous conclusion of the fourth voyage of Christopher Columbus (Rayes et al. 2015). Similarly, the burrowing activity of xylophagaids is reported to have caused the failure of early submarine telecommunications cables by damaging their insulating sheaths (Jeffries 1861; Parkes and Keeble 2016) and even today remains a concern for the design of subsea umbilicals (Parkes and Keeble 2016). Despite these negative impacts, wood-boring bivalves also play beneficial economic and ecological roles in many marine environments by converting recalcitrant wood into animal biomass that is more easily consumed by a wide range of organisms (Petra Pop et al. 2017; Cragg et al. 2020). Shipworms are also considered a delicacy in several traditional cuisines (Turner 1971) and have been proposed to have economic potential as a shellfishery (Willer and Aldridge 2020).

Despite their common xylotrophic lifestyle, these two families inhabit distinct and nearly nonoverlapping distributions wherein they are exposed to very different environmental conditions. Teredinidae are common in tropical and temperate waters but are largely absent from polar zones. With a few notable exceptions, for example (Velasquez and Shipway 2018), they occur at shallow depths, typically less than 200 m. They are most common in near-shore and intertidal, mangrove, estuarine, and coastal riparian environments where floating and deposited driftwood and submerged roots and branches provide abundant and consistent sources of food and shelter. Within these environments many teredinids experience and tolerate broad ranges of salinity and temperature with strong seasonal, diurnal, and weather-driven variation (Voight 2015). Because they often inhabit floating wood and wood deposited in the intertidal zone, they may frequently experience prolonged periods of air exposure due to tidal recession. They survive such exposure by sealing their burrows, which are lined with an impervious shell-like calcareous material, using a paired set of calcareous plates called pallets. Pallets and shell-lined burrows are common to Teredinidae but are absent in Xylophagaidae, with the exception of the genus *Xyloredo* in which the distal end of the burrow is lined (Voight et al. 2019). Although these adaptations may prevent desiccation and death during atmospheric exposure, they introduce other significant stresses such as extended periods of anaerobiosis and accumulation of metabolic waste.

Xylophagaidae, on the other hand, are found in sunken wood on the sea floor most commonly at depths greater than 150–200 m, with a few species coming into shallow water in boreal and high latitudes (Voight 2015; Romano et al. 2020). Typically, they are exposed to the consistent low temperatures and narrow salinity ranges characteristic of deep-sea environments and experience substantially less seasonal diurnal, and storm driven variation than is commonly encountered by teredinids in shallow water. With few exceptions, for example (Jayachandran et al. 2022), Xylophagaidae have not been reported to inhabit floating wood and have only rarely been observed within the intertidal zone (Turner 2002; Voight 2016). Thus, they are typically not exposed to the atmosphere, lack adaptations for sealing their burrows, and do not typically experience the types of anaerobic and metabolic stresses encountered by Teredinidae during prolonged periods of burrow closure.

The two families also differ with respect to reproductive strategies. Although little is known about the reproductive biology of Xylophagaidae, most are thought to be broadcast spawners with planktotrophic larvae. Although many species were once thought to brood their young, small specimens commonly found within the female’s burrows have more recently been shown to be dwarf males (Haga and Kase 2013) rather than larvae. Male dwarfism is often associated with sparse food availability and spatial limitation for growth (Haga and Kase 2013), features consistent with deep-water environments where wood, which serves both as food and habitat for Xylophagaidae, is scarce. In contrast, Teredinidae employ a wide range of reproductive strategies, including pseudocopulation (Shipway et al. 2020), broadcast spawning with maximized production of gametes, and larval brooding with fewer offspring and extended parental care. In most cases, these are adaptations suited to rapid utilization of a comparatively abundant but patchily distributed wood supply (MacIntosh et al. 2014). Male dwarfism is found in only one described teredinid species, *Zachsia zenkewitschi*, which inhabits spatially restrictive seagrass rhizomes (Shipway et al. 2016).

Finally, the two families differ substantially in anatomy and morphology. For the most part, Xylophagaidae conform to a typical bivalve body plan, wherein the internal organs are located between the anterior and posterior adductor muscles and the entire body, with the exception of the siphons in some cases, can be retracted between the shells (Voight 2015). In contrast, Teredinidae are among the most morphologically divergent bivalves (Turner 1966). During development, the body elongates and the heart, gills, gonads, and a substantial portion of the digestive system assume a position posterior to the posterior adductor muscle, bringing them permanently outside the protection of the valves.

**Fig. 2.**
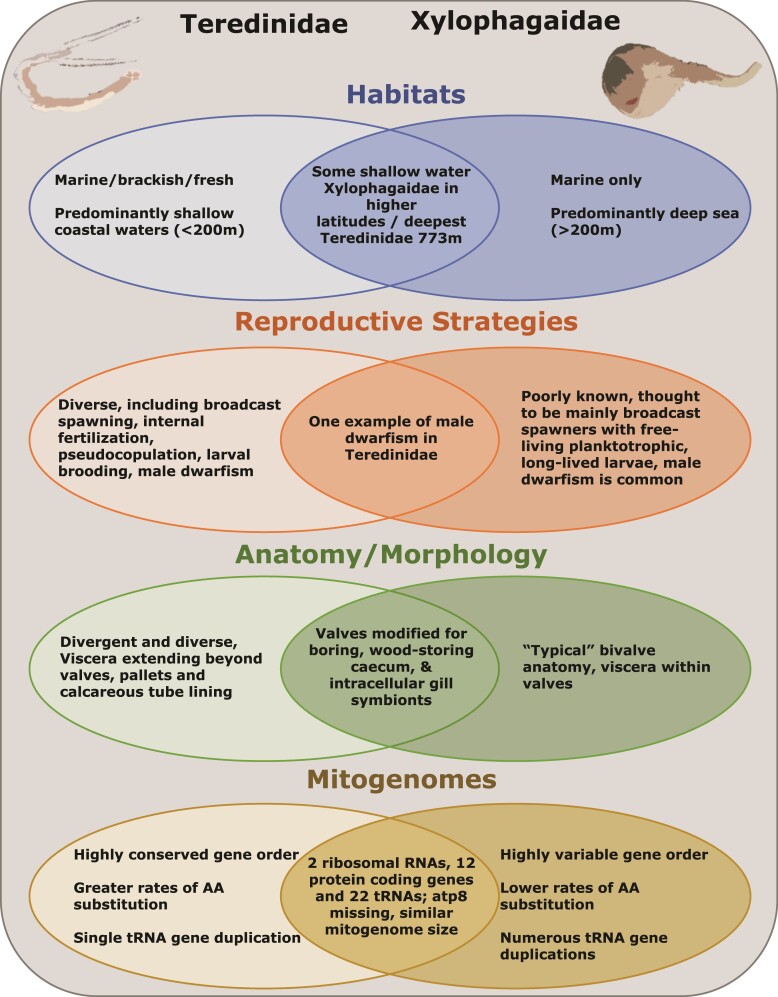
Graphic comparison of teredinid and xylophagaid habitat preferences, reproductive strategies, anatomy, and mitochondrial genome characteristics.

Despite these substantial differences (summarized in fig. 2), investigations based on 18S and 28S rRNA sequences support Teredinidae and Xylophagaidae as sister families (Voight 2015; Voight et al. 2019; Romano et al. 2020), agreeing with the earlier conclusions of Purchon (Purchon 1941) and supplanting the later placement of the deep-sea wood borers as a subfamily of Pholadidae (Turner 1967, 2002). Although no formal taxonomic revision has yet been published, this view has gained wide acceptance in the literature (Voight 2015; Voight et al. 2019; Romano et al. 2020). Nonetheless, phylogenetic relationships within the two families remain unresolved.

Mitochondrial genome data have proven useful in resolving phylogenetic relationships across a wide range of metazoans, for example (Miya et al. 2001; Osigus et al. 2013; Li, Kocot, et al. 2015) including mollusks, for example (Grande et al. 2008; Mikkelsen et al. 2018; Kong et al. 2020). Although, mollusks are among the most variable of bilaterian animals with respect to mitochondrial genome size and organization (Piccinini et al. 2021), most still conform to the canonical bilaterian mitochondrial genome complement of 37 genes including 22 tRNAs, 12–13 protein-coding genes, and 2 rRNA genes. ATP synthase subunit 8 is not detected and may be absent in a number of bivalve taxa (Ghiselli et al. 2021). The most obvious variations in length are due to the presence of noncoding regions (Formaggioni et al. 2021), which, in some bivalves, account for mitochondrial genomes 2–3 times of that of the typical 15–17 kb bilaterian size (Smith and Snyder 2007; Williams et al. 2017; Kong et al. 2020). Gene order, orientation, and location with respect to the heavy and light strands also show considerable variation in mollusks (Piccinini et al. 2021). However, the degree of variation in genome length, gene order, and strandedness, as well as nucleotide composition, appears to be largely clade-specific. In the case of the order Myida, which includes economically important invasive and wood-boring species, genomic resources are still scarce in comparison to other major bivalve clades. Prior to this study, only one complete mitochondrial genome assembly had been published for Myida in the NCBI Reference Sequence Database (O’Leary et al. 2016; Wilson et al. 2016) with no representation of the superfamily Pholadomyoidea, which includes most wood-boring species.

Because Xylophagaidae and Teredinidae present an unusual combination of recent common ancestry and unique shared feeding strategy, juxtaposed with profound differences in morphology, reproductive strategies, life history, and bathymetric distribution, we ask whether similarly stark contrasts are also observed in their mitochondrial genome organization and evolution. To this end, we explore the mitochondrial genomes from a variety of taxa representing both Teredinidae and Xylophagaidae.

## Results and Discussion

Mitochondrial genome sequences were determined for 42 bivalve specimens, including 26 Teredinidae from 15 locations and 16 Xylophagaidae from 8 locations (table 1). A single unique mitochondrial genome was recovered from each specimen examined. Although no evidence of distinct sex-specific mitochondrial lineages was detected, for example, doubly uniparental inheritance, this cannot be definitively ruled out as sex of individual specimens was not determined and reproductive organs were not specifically sampled.

**Table 1:** Information of Sample Collection Location

	Specimen	Collection location	Depth (m)	Coordinates
**Teredinidae**	*Bactronophorus thoracites* [PMS-2771X]	Infanta, Quezon, Philippines	<2	14.68367, 121.6369
	*Bactronophorus thoracites* [PMS-2849Y]	Infanta, Quezon, Philippines	<2	14.68367, 121.6369
	*Bankia* sp. [TBF03]	Pacoti River Environmental Protection Area, Ceara State, Brazil	<2	−3.84311, −38.42269
	*Bankia* sp. [TBF05]	Pacoti River Environmental Protection Area, Ceara State, Brazil	<2	−3.84311, −38.42269
	*Bankia gouldi* [5209S]	Mobile Bay, Gulf of Mexico, Alabama, USA	20	30.24867, −88.07333
	*Bankia setacea* [sp 1]	Puget Sound, Washington, USA	<20	47.85072, −122.33843
	*Bankia setacea* [sp 2]	Puget Sound, Washington, USA	<20	47.85072, −122.33843
	*Bankia setacea* [sp 3]	Puget Sound, Washington, USA	<20	47.85072, −122.33843
	*Bankia setacea* [sp 4]	Puget Sound, Washington, USA	<20	47.85072, −122.33843
	*Bankia setacea* [sp 5]	Puget Sound, Washington, USA	<20	47.85072, −122.33843
	*Dicyathifer mannii* [PMS-2772P]	Infanta, Quezon, Philippines	<2	14.68367, 121.6369
	*Dicyathifer mannii* [PMS-2858W]	Infanta, Quezon, Philippines	<2	14.68367, 121.6369
	*Dicyathifer mannii* [PMS-3770U]	Infanta, Quezon, Philippines	<2	14.68367, 121.6369
	*Kuphus polythalamius* [PMS-2132W]	Kalamansig, Sultan Kudarat, Philippines	<3	6.53631, 124.04836
	*Kuphus polythalamius* [PMS-2133X]	Kalamansig, Sultan Kudarat, Philippines	<3	6.53631, 124.04836
	*Kuphus* sp. [PMS-3700M]	Mabini, Batangas, Philippines	<2	13.75843, 120.92586
	*Lithoredo abatanica* [PMS-4316M sp 1]	Abatan River, Bohol, Philippines	<2	9.76558, 123.9442
	*Lithoredo abatanica* [PMS-4316M sp 2]	Abatan River, Bohol, Philippines	<2	9.765583, 123.9442
	*Lyrodus* sp. FLG0	Indian River Lagoon, Merritt Island, Florida, USA	<1	28.4060, 80.6603
	*Neoteredo reynei*	Coroa Grande Mangrove - Sepetiba Bay, Rio de Janeiro State, Brazil	<2	−22.90816, −43.87563
	*Nototeredo knoxi* [5147X]	Mobile Bay, Gulf of Mexico, Alabama, USA	20	30.24867, −88.07333
	*Teredo* sp. [TBF02]	Pacoti River Environmental Protection Area, Ceara State, Brazil	<2	−3.84311, −38.42269
	*Teredo* sp. [TBF07]	Pacoti River Environmental Protection Area, Ceara State, Brazil	<2	−3.84311, −38.42269
	*Teredo* sp. [TBF09]	Pacoti River Environmental Protection Area, Ceara State, Brazil	<2	−3.84311, −38.42269
	*Teredo bartschi*	Coos Bay, Oregon, USA	<2	43.325803, −124.20626
	*Teredothyra matocotana* [5007K]	Mobile Bay, Gulf of Mexico, Alabama, USA	20	30.24867, −88.07333
**Xylophagaidae**	*Xylonora corona*	BOWL3 (NE Pacific)	∼3,000	47.27, −127.59283
	*Xylonora zierenbergi*	BOWL3 (NE Pacific)	∼3,000	47.27, −127.59283
	*Xylophaga dorsalis*	Sørfjorden near Nygård, Norway	210	60.48191, 5.41775
	*Xylophaga oregona* [Bv346 11E]	BOWL6 (NE Pacific)	1,605	43.90866, −125.1715
	*Xylophaga oregona* [Bv346 18E]	BOWL6 (NE Pacific)	1,605	43.90866, −125.1715
	*Xylophaga oregona* [Bv352]	BOWL2 (NE Pacific)	1,596	47.957667, −126.0365
	*Xylophaga oregona* [Bv354]	BOWL2 (NE Pacific)	1,596	'47.957667, −126.0365
	*Xylophaga washingtona* [Dock3]	Friday Harbor Dock, Washington, USA	∼20	48.54485, −123.01231
	*Xylophaga washingtona* [Dock5]	Friday Harbor Dock, Washington, USA	∼20	48.54485, −123.01231
	*Xylophagaidae* sp. [E23]	Santos Basin (Brazil)	1,508	−25.90111, −45.035833
	*Xylophagaidae* sp. [sp1 E81]	Santos Basin (Brazil)	1,508	−25.90111, −45.03583
	*Xyloredo nooi* [E25]	Espirito Santo Basin (Brazil)	1,500	−21.45013, −39.8965
	*Xyloredo nooi* [E26]	Espirito Santo Basin (Brazil)	1,500	−21.45013, −39.8965
	*Xyloredo nooi* [E77]	Espirito Santo Basin (Brazil)	1,500	−21.45013, −39.8965
	*Xyloredo* sp. [E88]	Santos Basin (Brazil)	3,358	−28.03638, −43.53833
	*Xyloredo* sp. [E89]	Santos Basin (Brazil)	3,358	−28.03638, −43.53833

**Fig. 3.**
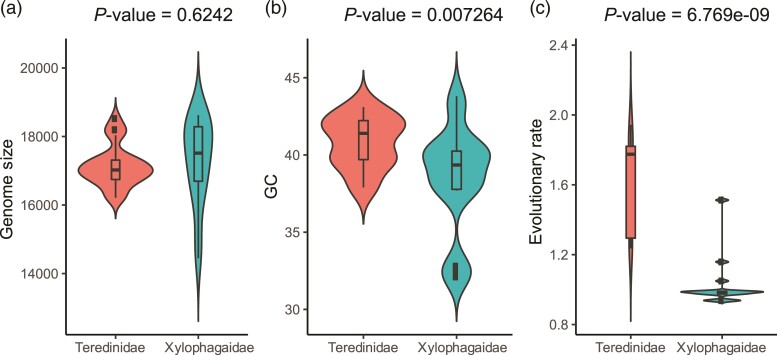
Violin plots comparing genome size, GC-content, and evolutionary (amino acid substitution) rate between Teredinidae and Xylophagaidae. Statistical significance of differences between Teredinidae and Xylophagaidae with respect to each genome property was evaluated using paired *t*-tests with *P* values adjusted by Bonferroni methods (*P* values shown above). (*a*) Genome size; (*b*) GC content; and (*c*) evolutionary (amino acid substitution) rates as measured by the tip-to-root distance. Note that GC-content and amino acid substitution rate, but not genome size, are significantly different at a *P* value threshold of <0.01.

All mitochondrial genomes described herein contain, at minimum, 2 ribosomal RNA (rRNA) genes, 22 tRNA genes, and 12 of the 13 canonical protein-coding genes commonly present in animal mitogenomes; *atp8* was not detected. All genes are encoded on the same strand and share the same orientation. The genomes range in size from 14,450 to 18,624 bp (table 2), similar to most bilaterian mtDNA genomes. No significant difference was detected in mitochondrial genome size between the two families. Much of the observed length variation is associated with noncoding regions, which on average comprised a significantly larger fraction of total mitochondrial genome in Xylophagaidae than in Teredinidae (supplementary fig. S1, Supplementary Material online). However, significant differences were observed in GC-content and in evolutionary rates between teredinids and xylophagaids using paired *t*-tests with *P* values adjusted by Bonferroni methods (fig. 3).

**Table 2:** Genome Statistics

	Specimen	Genome size (bp)	GC%	Sequence platform	GenBank accession #
**Teredinidae**	*Bankia setacea* [sp 1]	16,986	39.8	Illumina, 454 GS FLX Titanium	OM910805
	*Bankia setacea* [sp 2]	16,879	39.9		OM910806
	*Bankia setacea* [sp 3]	17,025	39.7		OM910807
	*Bankia setacea* [sp 4]	17,105	39.8		OM910808
	*Bankia setacea* [sp 5]	17,358	40.0		OM910809
	*Bankia* sp. [TBF03]	16,411	41.4	Illumina MiSeq	OM910810
	*Bankia* sp. [TBF05]	16,766	41.4		OM910811
	*Neoteredo reynei*	18,035	41.5		OM910821
	*Teredo* sp. [TBF02]	17,016	41.7		OM910824
	*Teredo* sp. [TBF07]	17,022	41.7		OM910825
	*Teredo* sp. [TBF09]	17,028	41.7		OM910826
	*Teredo bartschi*	16,962	39.9	Illumina HiSeq X	OM910823
	*Bactronophorus thoracites* [PMS-2771X]	16,562	43.1	Illumina HiSeq 2000	OM910802
	*Bactronophorus thoracites* [PMS-2849Y]	16,562	43.1		OM910803
	*Bankia gouldi* [5209S]	16,795	39.0		OM910804
	*Dicyathifer mannii* [PMS-2772P]	17,176	37.9		OM910812
	*Dicyathifer mannii* [PMS-2858W]	17,176	37.9		OM910813
	*Dicyathifer mannii* [PMS-3770U]	17,177	37.9		OM910814
	*Kuphus polythalamius* [PMS-2132W]	18,094	42.4		OM910815
	*Kuphus polythalamius* [PMS-2133X]	18,098	42.3		OM910816
	*Kuphus polythalamius* [PMS-3700M]	18,578	42.6		OM910817
	*Lithoredo abatanica* [PMS-4316M sp 1]	16,084	42.3		OM910818
	*Lithoredo abatanica* [PMS-4316M sp 2]	16,074	42.3		OM910819
	*Lyrodus* sp. [FL G0]	17,907	37.7		OM910820
	*Nototeredo knoxi* [5147X]	18,431	39.7		OM910822
	*Teredothyra matocotana* [5007K]	17,364	39.7		OM910827
**Xylophagaidae**	*Xylonora corona*	15,083	42.9	Illumina HiSeq 2000	OM910828
	*Xylonora zierenbergi*	14,450	43.8		OM910829
	*Xylophaga dorsalis*	16,787	32.8		OM910830
	*Xylophaga oregona* [Bv346 11E]	18,220	40.2		OM910831
	*Xylophaga oregona* [Bv346 18E]	17,883	40.2		OM910832
	*Xylophaga oregona* [Bv352]	18,477	40.4		OM910833
	*Xylophaga oregona* [Bv354]	18,477	40.4		OM910834
	*Xylophaga washingtona* [Dock3]	18,624	40.0		OM910835
	*Xylophaga washingtona* [Dock5]	18,599	40.0		OM910836
	*Xylophagaidae* sp. [E23]	17,410	38.7		OM910837
	*Xylophagaidae sp.* [sp1 E81]	16,921	38.3		OM910838
	*Xyloredo nooi* [E25]	17,619	37.8		OM910839
	*Xyloredo nooi* [E26]	18,013	38.1		OM910840
	*Xyloredo nooi* [E77]	17,322	37.7		OM910841
	*Xyloredo* sp. [E88]	16,184	32.1		OM910842
	*Xyloredo* sp. [E89]	16,416	32.4		OM910843

### Phylogeny

**Fig. 4.**
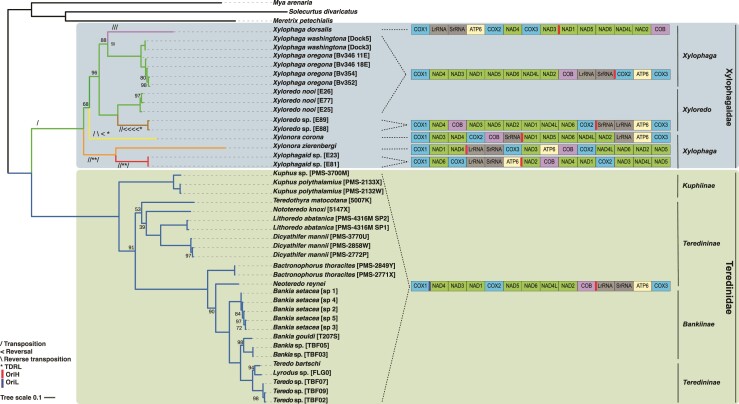
Phylogenetic relationships and variation in mitochondrial gene arrangement among species of Xylophagaidae and Teredinidae. Phylogenetic tree inferred by ML (single-site homogeneous model, unpartitioned, IQ-Tree 1.6.7) based on 4,135 unambiguously aligned amino acid positions selected using Gblocks from an alignment of 12 concatenated mitochondrial protein-coding genes. Bootstrap proportions, less than 100 are indicated at the nodes; where no numerical value is indicated, the bootstrap proportion = 100. Protein-coding gene arrangements associated with each species are depicted on the right. Putative origins of replication are indicated by thick vertical bars. A proposed scenario for the evolutionary history of gene rearrangement, determined by analysis of common intervals using CRex, is superimposed on the phylogenetic tree with branches color-coded according to extant and hypothetical ancestral gene orders. Symbols indicate rearrangements required to transform each hypothetical ancestral gene order to the order observed in the extant species. “/” indicates  transposition, “\” indicates reverse transposition, “<” indicates reversal, “*” indicates tandem duplication with random loss (TDRL).

All three modeling approaches (single-site homogeneous model of unpartitioned supermatrix (fig. 4), site-heterogenous C20 model (supplementary fig. S4, Supplementary Material online), and data-partitioning (supplementary fig. S5, Supplementary Material online), produced largely congruent results, with exception of the phylogenetic positions of *Teredothyra matocotana* and *Nototeredo knoxi* for which bootstrap support was low in all three analyses. The site homogeneous model applied to the unpartitioned supermatrix produced a tree most similar to previously published phylogenetic analyses based on large (28S) and small (18S) subunit nuclear-encoded rRNA sequences and the cytochrome c oxidase I gene (Distel et al. 2011; Voight et al. 2019; Romano et al. 2020). For example, in agreement with previous reports, the tree presented in figure 4 supports the nonmonophyly of the Teredinidae subfamilies Teredininae and Bankiinae, showing *Lyrodus* and *Teredo* (Teredininae) nested within Bankiinae, which is in turn nested within a clade containing *Neoteredo* and *Bactronophorus* (Teredininae). Additionally, in agreement with previously published results, these analyses support the monophyly of *Xyloredo* and of *Xylophaga* as well as the divergence between these and *Xylonora*, a genus recently erected to remedy the nonmonophyly of *Xylophaga* (Voight et al. 2019; Romano et al. 2020). However, the monophyly of *Xylonora* is not well supported in our analyses. The most notable difference between the analyses presented here and those reported previously (Distel et al. 2011; Borges et al. 2022) is the basal position of the node connecting *Kuphus* (Kuphiinae) to other Teredinidae, which was previously reported to be nested within Teredininae. As *Kuphus* is arguably the most morphologically derived and physiologically distinct member of Teredinidae, as well as one of only two members of the family identified to date that harbor chemoautotrophic endosymbionts (Distel et al. 2017; Altamia et al. 2020), the phylogenetic position of Kuphiinae is important with respect to understanding the origins of symbiosis in teredinids. The tree is also consistent with the previously reported sister relationship between the families Teredinidae and Xylophagaidae (Distel et al. 2011). We caution, however, that the analyses presented here lack sufficient taxonomic representation both within the two families and among their closest relatives to confidently resolve the basal branching order of either Teredinidae or Xylophagaidae, or to provide additional support for the proposed sister relationship between these taxa. Based on the remarkable economic impact, ecological importance, historical influence, and potential biotechnological and medicinal relevance of these organisms, future studies should seek to analyze mitogenomes across a broader taxonomic range of both families.

### Gene Order and Copy Number

Surprisingly, despite their close phylogenetic relationship, the two families differ dramatically with respect to conservation of gene order. Among all Teredinidae examined, the order of protein-coding genes in mitochondrial genomes is conserved (fig. 4), but variation was observed among tRNA positions (supplementary fig. S6, Supplementary Material online). In 7 of 14 taxa, 1–4 tRNA genes (*trn L1, M, N, Q, R,* and *V*) differ in position from the consensus order. This is consistent with the observation that rearrangements and duplications involving tRNAs occur more frequently than those observed among protein-coding genes (Ghiselli et al. 2021). Interestingly, in one species, *Neoteredo reynei*, there is an apparent duplication of *trnW* and its consensus neighbor, *cox3*. E values and quality scores determined by MITOS2 suggest the degradation of one copy of each duplicated gene.

In sharp contrast, six distinct and highly divergent patterns of protein-coding gene order were observed among eight putative xylophagaid species examined (fig. 4). In addition, variations in tRNA gene order are far more common in Xylophagaidae than in Teredinidae, as are gene duplications (supplementary fig. S6, Supplementary Material online). Indeed, differences in tRNA gene order were apparent between all putative species examined and were observed even within a single nominal species, *Xylophaga oregona*. Apparent tRNA gene duplications were observed in three of eight putative species examined, involving *trnS1, H, D, Y, Q,* and *M*, with putative copy numbers ranging from 2 to 5 per genome. Similarly multiple tRNA gene copies have been reported on other bivalve mitochondrial genomes, for example, in bathymodiolin mussels (Zhang et al. 2021). Moreover, in one putative species, represented by xylophagaid specimens E23 and E81, there is an apparent tandem duplication of the *rrnS* and *trnM* genes. Although tandem duplication is thought to play an important role in mitochondrial genome rearrangement, few animal mitochondrial genomes contain duplicated copies of protein-coding and ribosomal genes, likely due to selection for the maintenance of cytonuclear stoichiometry (Ghiselli et al. 2021). Thus, the evidence of gene duplications presented here warrant further investigation.

Interestingly, among all pairwise comparisons within and between the two families, the most similar arrangements of mitochondrial protein-coding genes are observed between all Teredinidae and *Xylophaga washingtona*, *X. oregona* and three of five *Xyloredo* mitogenomes examined (fig. 4). These two protein-coding gene arrangements differ only by the position of the *cox2* gene, and each can be transformed into the other with a single transposition. This observation, combined with the complete conservation of protein-coding gene order in Teredinidae, suggest the parsimonious conclusion that a similar protein-coding gene arrangement likely occurred in the most recent common ancestor of the two families. Based on this assumption, credible scenarios for evolution of gene order can be inferred by analysis of common intervals using Crex (Bernt et al. 2007). For simplicity, the scenario depicted in figure 4 considers only protein-coding gene order and proposes an ancestral gene order identical to that found in all Teredinidae. Although resolving the complex history of mitochondrial gene rearrangement in wood-eating taxa requires additional taxon sampling and is beyond the scope of this investigation, the scenario presented in figure 4 serves to demonstrate the large number and diverse types of rearrangements required to reconcile the numerous and divergent gene orders observed in this investigation. Notably, protein-coding gene rearrangements are found even among closely related members of the genus *Xyloredo*, with some members sharing the same gene order as *X. washingtona* and *X. oregona* and others displaying a highly divergent gene order.

In addition to greater conservation of gene order, species of Teredinidae also displayed greater conservation with respect to the predicted locations of replication origins (table 3). Within Teredinidae, the predicted locations of both the heavy-strand origins (OriH) and light-strand origins (OriL) were conserved with respect to protein-coding genes but not with respect to tRNA genes. In all species the predicted OriH was located between *cob* and *rrnL*. However, the predicted OriH locations differed with respect to the locations of trnM and trnC which, in all but one species also fall between these same protein-coding genes. The predicted OriH in all but three taxa (*Bactronophorus thoracities, Dicyathifer mannii* and *Lithoredo abatanica*), falls downstream of *cob* but precedes trnM and trnC. In *B. thoracities*, trnM is transposed to a position upstream of *cob* and the OriH falls between *cob* and trnC. In *L. abatanica* the predicted OriH falls beween trnM and trnC, and in *D. mannii* it falls between trnM and *rrnL.* Similarly, the predicted light-strand origin (OriL) was located between *cox1* and *nad4* in all species but differed with respect to the position of *trnT*, which also falls between these two protein-coding genes. The OriL is predicted to fall in the *cox1*-trnT IGR in all teredinid mitogenomes except *D. mannii* and *L. abatanica* in which it falls downstream of trnT. Thus, the locations of both origins of replication appear to be invariant with respect to protein-coding genes but variable with respect to tRNA encoding genes.

**Table 3:** Hypothesized Origins of Replication in Teredinid and Xylophagaid Mitogenomes as Revealed by a DNAWalk Analysis

	OriH	OriL
**Teredinidae**		
*Bactronophorus thoracites*	*cob*-trnM IGR	*cox1*-trnT IGR
*Bankia gouldi*	*cob*-trnM IGR	*cox1*-trnT IGR
*Bankia setacea*	*cob*-trnM IGR	*cox1*-trnT IGR
*Bankia* sp.	*cob*-trnM IGR	*cox1*-trnT IGR
*Dicyathifer mannii*	trnC-16S IGR	trnT*-nad4* IGR
*Kuphus polythalamius*	*cob*-trnM IGR	*cox1*-trnT IGR
*Lithoredo abatanica*	trnM-trnC IGR	*trnT-nad4* IGR
*Lyrodus* sp.	*cob*-trnM IGR	*cox1*-trnT IGR
*Neoteredo reynei*	*cob*-trnM IGR	*cox1*-trnT IGR
*Nototeredo knoxi*	*cob*-trnM IGR	*cox1*-trnT IGR
*Teredo bartschi*	*cob*-trnM IGR	*cox1*-trnT IGR
*Teredo* sp.	*cob*-trnM IGR	*cox1*-trnT IGR
*Teredothyra matocotana*	trnC-16S IGR	*cox1*-trnT IGR
		
**Xylophagaidae**		
*Xylonora corona*	12S-trnI IGR	ND
*Xylonora zierenbergi*	*nad4*-trnE IGR	ND
*Xylophaga dorsalis*	trnK-trnL2 IGR	ND
*Xylophaga oregona*	trnR-*cox2* IGR	ND
*Xylophaga washingtona*	trnR-*cox2* IGR	ND
*Xylophagaid* sp.	trnC-trnI IGR	ND
*Xyloredo nooi*	trnR-*cox2* IGR	ND
*Xyloredo* sp.	trnS1-trnY IGR	ND

IGR, intergenic region; ND, not determined; OriH, heavy-strand origin of replication; OriL, light-strand origin of replication; trn, transfer RNA.

In contrast, among the eight species Xylophagaidae examined, six distinct locations were predicted for the OriH with respect to flanking protein-coding genes. Interestingly, among xylophagaid species that share the same protein-coding gene order (*Xylophaga oregona, X. washingtona* and *Xyloredo nooi*) the predicted OriH locations were conserved with respect to flanking protein-coding genes. The position of OriL could not be confidently predicted in the examined xylophagaid species.

### Amino Acid Substitution Rates

In contrast to the rapid evolution of gene order in Xylophagaidae as compared Teredinidae, the estimated average rate of amino acid substitution (1.53 substitutions/site), determined as a function of tip-to-root branch lengths in the concatenated supermatrix tree (figure 4), is significantly lower (*P* = 6.7 × 10^−9^, *t*-test) than in Teredinidae (2.18 substitutions/site; fig. 3). This constitutes a difference of approximately 1.4-fold. The higher rates of amino acid substitution in Teredinidae may be tied to adaptation to anaerobic stress. Unlike xylophagaids which are adapted to deep-sea environments where oxygen concentrations are comparatively consistent and predictable, teredinids are adapted to shallow water, intertidal environments, and floating wood, where exposure to the atmosphere and subsequent desiccation is a significant threat. To combat desiccation, teredinids seal their burrows with their paired pallets, allowing some species to survive for weeks in wood removed from water, but also exposing them to extended periods of anoxia (Lane et al. 1955).

Also interesting is the observation that within Teredinidae, the average amino acid substitution rate (2.34 substitutions/site) estimated for the clade containing the genera *Bactronophorus*, *Neoteredo*, *Bankia, Teredo* and *Lyrodus* is significantly higher (*P* = 3.5 × 10^−8^, Kruskal-Wallis H-test) than that estimated for the remaining teredinid mitogenomes (1.84 substitutions/site) (supplementary fig. S7, Supplementary Material online). Several unusual reproductive strategies are observed within this clade. Although the details of reproductive behavior are not known in *Bactronophorus* and *Neoteredo*, internal fertilization is observed in *Bankia, Teredo, and Lyrodus*. In *Bankia* species, pseudocopulation is known to occur, in which sperm is delivered from the exhalent siphon of one individual to the inhalant siphon of a neighbor (Shipway et al. 2020). After internal fertilization and before release to the environment, species of *Teredo* and *Lyrodus* brood their young to late larval stages in pouches located on the dorsal side of the gill. Pseudocopulation and larval brooding favor more localized reproduction and more limited larval dispersal. These taxa are also generalists that inhabit many types of floating wood, as opposed to other teredinids that tend to inhabit less mobile habitats such as mangrove roots, sulfidic sediments (Distel et al. 2017) and limestone riverbanks (Shipway, Distel, et al. 2019; Shipway, Rosenberg, et al. 2019). Finally, all of these taxa are hermaphroditic and at least some are known to be capable of self-fertilization (Eckelbarger and Reish 1972). These strategies are likely adaptive for utilization of wood that is often patchily distributed in marine environments. They promote efficient localized settlement on and consumption of floating wood that may be rafted great distances from other wood sources (Treneman, Borges, et al. 2018; Treneman, Carlton, et al. 2018). Additionally, these strategies allow few or even single larvae to initiate new populations on wood islands. However, these same adaptations may also result in genetic bottlenecks resulting from the frequent transport and isolation of small founding populations, which in turn can promote the fixation of mutations. Indeed, this notion is supported by the observed prevalence of cryptic species in some of these taxa (Borges and Merckelbach 2018).

Unfortunately, relatively little is known about reproductive strategies in Xylophagaidae. However, a recent study used dynamic energy budget modeling to propose a different strategy for adaptation to life on sparse and patchy wood islands in *Xylonora atlantica*, involving rapid sexual maturation at small adult body size, a long-lived larval dispersal stage and high larval survival rates (Gaudron et al. 2021). If common to other Xylophagaidae, this strategy might be less prone to geographic isolation and genetic bottlenecks, leading to greater effective population sizes and more efficient purifying selection.

## Conclusions

Identifying causal relationships between the functional and physiological adaptations of taxa and the observed differences in their mitochondrial genome organization and evolution is notoriously difficult (Ghiselli and Milani 2020; Ghiselli et al. 2021). Here, we show that two bivalve sister clades, Teredinidae and Xylophagaidae, have followed very different evolutionary trajectories, one leading to thriving existence in comparatively warm productive surface waters and diverse coastal habitats and the second to considerable success on the comparatively cold, relatively uniform and oligotrophic deep-sea floor. Teredinidae includes very diverse species that are known to thrive in a broad and variable range of environmental conditions and that span a wide range of life histories, feeding and reproductive strategies, and physiologies. Unfortunately, the parallel properties with respect to Xylophagaidae are less well-known, making generalization more difficult. Although we can infer differences based on their distinct respiratory demands, reproductive strategies, bathymetric ranges and the environmental variables that characterize these distributions, distinguishing between these alternatives, or other explanations will require more taxon sampling and better understanding of wood-boring bivalve phylogeny and ecology. Perhaps the most interesting feature of this data set is the contrasting patterns observed in the two families with respect to rates of sequence evolution and rates of genome rearrangement, phenomena which previously have been proposed to be positively correlated (Mortz et al. 2021). While we cannot currently explain these differences, the presented data provide fertile ground for exploration of the environmental, biological, and molecular mechanisms that shape the tempo and mode of mitochondrial evolution.

## Materials and Methods

### Specimen Collection and DNA Extraction

Specimens were collected by a variety of methods ranging from collection by hand, to dredging, and deployment and recovery of wooden substrates (table 1). Specimens were frozen at −80 °C or preserved in 80–100% nondenatured ethanol following collection unless specified otherwise. For Xylophagaidae, siphon tissue was dissected from each bivalve, and total genomic DNA was extracted using the DNeasy Blood & Tissue Kit (Qiagen) according to the manufacturer’s protocols. Tissue samples collected in Alabama and Florida were preserved in 0.25 M EDTA, pH 8.0 (Sharpe et al. 2020). Total genomic DNA was extracted using the DNeasy Blood & Tissue Kit (Qiagen) and concentrated using the DNA Clean & Concentrator-25 Kit (Zymo Research) following manufacturer’s recommended protocols.

### Mitochondrial Genome Sequencing, Assembly, and Annotation

Sequencing platforms and GenBank submission information are outlined in table 2. For Xylophagaidae, sequencing of genomic DNA was performed by The Genomic Services Lab at the Hudson Alpha Institute in Huntsville, Alabama using Illumina (San Diego, California, USA) 2 × 150 paired-end TruSeq protocols on an Illumina HiSeq 2500 platform. The paired-end reads from each of the operational taxonomic units (OTUs) were assembled de novo using Ray 2.3.1 (Boisvert et al. 2010) with k-mer = 31. For Teredinidae, except *Teredo bartschi* and *Bankia setacea,* mitochondrial genomes were extracted from gill tissue metagenome assemblies that contained both host and symbiont DNA and that were sequenced using Illumina HiSeq 2000 sequencer with 350-bp inserts and 125-bp paired-end reads at the Huntsman Cancer Institute’s High-Throughput Genomics Center at the University of Utah. Illumina fastq reads were trimmed using Sickle v1.33 (Joshi and Fass 2011), merged, and converted to FASTA files. Merged FASTA files were assembled using IDBA_ud v2.0 (Peng et al. 2012) using default parameters. *Teredo* sp. and *Bankia* sp. gill metagenomes were sequenced using Illumina MiSeq. The raw reads were assembled using either the metaspades pipeline of SPAdes (version 3.11.1) (Bankevich et al. 2012) or IDBA-UD (version 2) (Peng et al. 2012). Before assembly, raw reads were merged using BBMerge (v9.02) (Bushnell et al. 2017). Nonmerged reads were filtered and trimmed using FaQCs (Version 1.34) (Wang et al. 2014). Mitochondrial contigs were identified using TBLASTN (Altschul et al. 1997) and the previously published bivalve mitochondrial genome from *Mya arenaria* (Wilson et al. 2016) as bait. For *T. bartschi*, whole genome shotgun (WGS) sequencing was conducted at the New York Genome Center on an Illumina HiSeqX (2 × 150 bp). Library preparation utilized a TruSeq PCR-free kit (450 bp). The mitochondrial genome was bioinformatically extracted from the WGS run using MitoFinder v1.4 (Allio et al. 2020). MitoFinder employed MEGAHIT (Li, Liu, et al. 2015) for mitogenome assembly and tRNAscan-SE (Chan and Lowe 2019) for tRNA annotation. Annotation of the mitochondrial genomes was conducted initially with MITOS2 web server (Bernt et al. 2013) with default settings and the invertebrate genetic code (i.e., NCBI translation table 5) for mitochondria, followed by manual genome annotation of start and stop positions of each gene using Artemis (Rutherford et al. 2000). The nucleotide base composition across the complete mitochondrial genome, protein-coding gene sequences and 3rd codon position were calculated using Artemis.

### Phylogenetic Methods

Forty-two specimens were included in the phylogenetic analysis (table 1), including 26 Teredinidae and 16 Xylophagaidae. *M. arenaria* (Myida), *Meretrix petechialis* (Venerida) and *Solecurtus divaricatus* (Cardiida) were selected as the outgroup for our phylogenetic analysis based on availability in the NCBI RefSeq (O’Leary et al. 2016) database and the current understanding of bivalve evolutionary relationships (Combosch et al. 2017; Lemer et al. 2019). Prior to alignment, nucleotide sequences were translated to amino acids using invertebrate mitochondrial genetic translation code as implemented in Artemis. Each protein-coding gene was individually aligned in MAFFT v7.2.3 (Katoh and Standley 2013) followed by manual correction. Each gene was trimmed using Gblocks (Talavera and Castresana 2007) to discard ambiguously aligned sites with default parameters. Protein-coding gene alignments were then concatenated into a final supermatrix dataset using FASconCAT (Kuck and Meusemann 2010).

For the supermatrix constructed above, we used three different approaches to infer the shipworm phylogeny: 1) the concatenation (i.e., supermatrix) approach with a single site-homogeneous model or partition, 2) the concatenation approach with data-partitioning by gene, and 3) the concatenation approach with a site-heterogenous C20 model to account for amino acid compositional heterogeneity. Maximum-likelihood (ML) phylogenetic analysis was conducted using IQ-Tree 1.6.7 (Nguyen et al. 2015). Prior to ML analyses, ModelFinder (Kalyaanamoorthy et al. 2017) was used to evaluate best substitution models for each gene partition. Nodal support for ML analyses was evaluated with 1,000 ultrafast bootstrapping replicates. Note that only bootstrap support ≥95 should be considered as a strong support for a given bipartition (Minh et al. 2013).

### Mitochondrial Genome Properties

We focused our analyses on the comparisons of five mitochondrial genome properties between Xylophagaidae and Teredinidae. Specifically, for a given taxon, 1) amino acid substitution rate was estimated as the distance from the most recent common ancestor shared by Xylophagaidae and Teredinidae to each tip on the concatenation-based supermatrix ML tree; 2) GC-content was calculated as the percentage of G and C nucleotides in the complete genome; 3) gene order was determined based on analysis of gene annotations predicted using MITOS 2.0 (Donath et al. 2019); 4) genome size was determined as the number of base pairs per genome, and 5) percent coding sequence was estimated as the number of nucleotides in coding sequences divided by the total genome size 100×. To determine whether there was a significant difference of properties between Xylophagaidae and Teredinidae, we conducted a paired *t*-test with value adjusted by Bonferroni methods using R package rstatix (Team 2021). Branch length differences among subtrees were examined using the Kruskal–Wallis H-test. Scenarios for potential gene rearrangements were explored based on analysis of common intervals using Crex (Bernt et al. 2007) as implemented on the MITOS2 server. The DNA Skew Graphing tool (Thomas et al. 2007), available online via the Viral Bioinformatics Research Centre (https://4virology.net/), was used to search representative mitochondrial genomes for abrupt changes in base composition bias that are characteristic of both the heavy-strand origin of replication (OriH) and light-strand origin of replication (OriL). After locating putative origins of replication, we utilized the UNAFold web server (Zuker 2003) to locate stable stem-loop configurations containing characteristic T-rich loops (see supplementary figs. S2 and S3, Supplementary Material online; also (Brugler and France 2008) for a list of features typically associated with origins of replication and the application of the “DNA Walker” (Lobry 1996) graphing option to locate putative Oris within a mitogenome).

## Supplementary Material

Supplementary data are available at *Genome Biology and Evolution* online.

## Supplementary Material

evac089_Supplementary_Data

## Data Availability

The mitochondrial genome sequences described in this manuscript have been submitted to the Genbank database (NCBI). Accession numbers are listed in table 2. Other datasets generated and/or analyzed during the current study are available in the Figshare repository, https://figshare.com/s/e0824b4f237b54765717.
