# Supplementary material for: Contrasting Modes of Mitochondrial Genome Evolution in Sister Taxa of Wood-Eating Marine Bivalves (Teredinidae and Xylophagaidae)
Source: Genome Biol Evol. 2022 Jun 17;14(6):evac089. doi: 10.1093/gbe/evac089 (PMC9226539; doi:10.1093/gbe/evac089)
Supplement: evac089_Supplementary_Data [file evac089_supplementary_data.docx]

**Contrasting modes of mitochondrial genome evolution in sister taxa of wood-eating marine bivalves (Teredinidae and Xylophagaidae).**

Yuanning Li, Marvin A. Altamia, J. Reuben Shipway, Mercer R. Brugler, Angelo Fraga Bernardino, Thaís Lima de Brito, Zhenjian Lin, Francisca Andréa da Silva Oliveira, Paulo Sumida, Craig R. Smith^1^, Amaro Trindade-Silva, Kenneth M. Halanych & Daniel L. Distel

**Supplemental Figures**

**Supplemental Figure S1. Violin plots comparing average percent coding sequence per genome for Teredinidae and Xylophagaidae.** Statistical significance of the difference between Teredinidae and Xylophagaidae was evaluated using paired *t*-tests with *P* values adjusted by Bonferroni methods (*P* values shown above). Note that duplicate copies of genes were considered as non-coding sequence in this analysis on the presumption that they are non-functional.


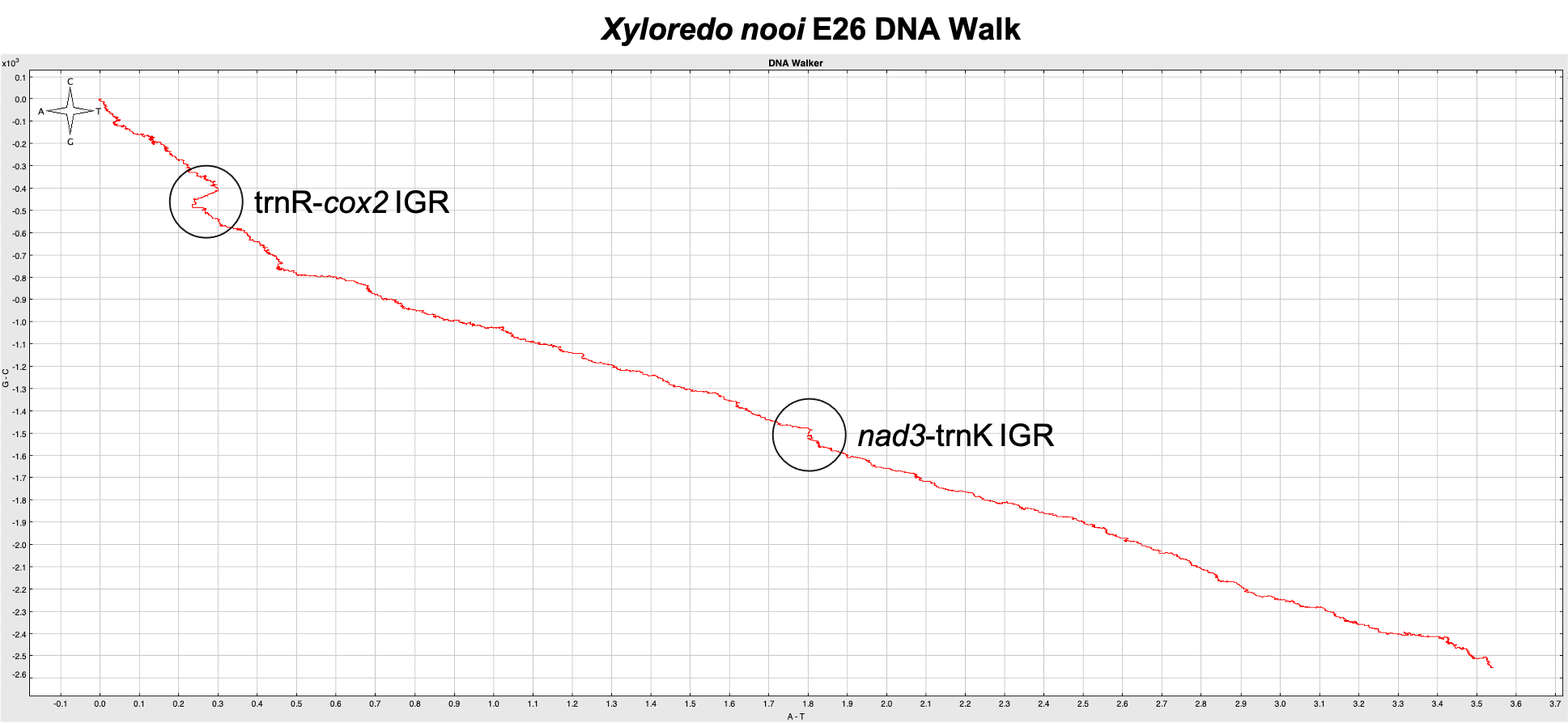


**Supplemental Figure S2.**A DNA Walk along the mitochondrial genome of *Xyloredo nooi* (E26). Each step in the walk proceeds in a direction determined by base composition (see compass in upper left). Circles highlight abrupt changes in base composition bias that are typically associated with origins of replication. The intergenic region (IGR) between *trnR* and *cox2* was identified as the heavy strand origin of replication while the IGR between *nad3* and *trnK* was putatively identified as the light strand origin.

**Supplemental Figure S3.**A stem-loop structure located in an intergenic region (IGR) between *cob* and trnM in the mitogenome of *Bankia gouldi* (5262M). The *cob*-trnM IGR was identified as the heavy strand origin of replication. The long stem is stabilized by G-C bonds and the characteristic loop is rich in T nucleotides.

**Supplemental Figure S4. Phylogenetic relationships among species of Xylophagaidae and Teredinidae using site-heterogeneous model.** Phylogenetic tree inferred by maximum likelihood (C20 model, IQ-Tree 1.6.7.) based on 4,135 unambiguously aligned amino acid positions selected using Gblocks from an alignment of 12 concatenated mitochondrial protein coding genes. Bootstrap proportions, less than 100 are indicated at the nodes; where no numerical value is indicated, the bootstrap proportion = 100.

**Supplemental Figure S5. Phylogenetic relationships among species of Xylophagaidae and Teredinidae using partition by genes.** Phylogenetic tree inferred by maximum likelihood (C20 model, IQ-Tree 1.6.7.) based on 4,135 unambiguously aligned amino acid positions selected using Gblocks from an alignment of 12 concatenated mitochondrial protein coding genes. Bootstrap proportions, less than 100 are indicated at the nodes; where no numerical value is indicated, the bootstrap proportion = 100.


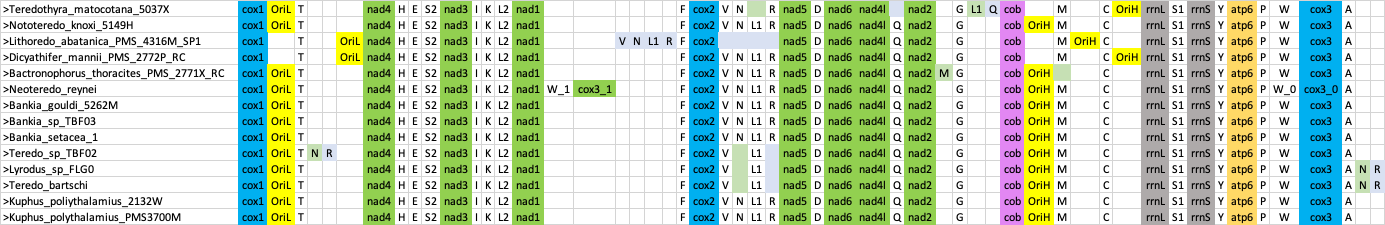


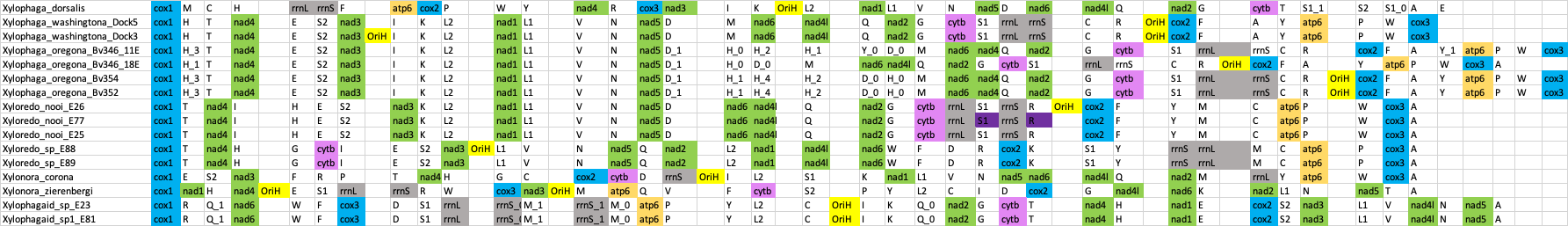


**Supplemental Figure S6.** **Detail of gene order and content for examined members of Teredinidae (above) and Xylophagaidae (below).** Gene annotations were performed using MITOS2 web server (Bernt, et al. 2013) (Ghiselli, et al. 2021) Blue, cytochrome c oxidase subunits 1-3; green, nicotine adenine nucleotide dehydrogenase subunits 1-6; purple, cytochrome b; grey, large and small subunit ribosomal RNAs; gold, ATP synthase subunit 6; yellow, heavy and light strand origins of replication; dark purple, genes not detected; light blue and light green indicate deviations from the consensus gene order in Teredinidae. Putative gene duplicates are numbered in order of increasing E values/decreasing annotation quality scores as determined by MITOS2.

**Supplemental Figure S7. Violin plots comparing amino acid substitution rates between Xylophagaidae and two Teredinidae subtrees.** Rates were estimated as root-to-tip branch lengths for each specimen, inferred using the single site-homogeneous model, unpartitioned, IQ-Tree 1.6.7. Teredinidae group 1 contains the subtree including the genera *Bactronophorus, Neoteredo, Bankia, Lyrodus and Teredo.* Teredinidae group 2 contains all remaining Teredinidae examined. Statistical significance of differences between groups was determined using the Kruskal-Wallis H-Test. P-values are displayed above the brackets and the global P-value is displayed at the top.
